# Humanized Mouse Models of Epstein Barr Virus Infection

**DOI:** 10.1002/cpz1.70257

**Published:** 2025-11-25

**Authors:** Saskia Gertrud von Boxberg, Kristin Gehrmann, Svenja Luisa Nopper, Lucas Romann, Svenja Kösegi, Christian Münz

**Affiliations:** ^1^ Viral Immunobiology, Institute of Experimental Immunology University of Zürich Zürich Switzerland

**Keywords:** B‐cell lymphoma, Epstein Barr virus (EBV), human immune system reconstitution, immunocompromised mice, natural killer (NK)‐cell responses, T‐cell responses, xenograft

## Abstract

The oncogenic Epstein Barr virus (EBV) is an exclusively human pathogen with related lymphocryptoviruses (γ1‐herpesviruses) only present in monkeys. Therefore, experimentation with EBV infection in a small animal model requires reconstitution or adoptive transfer of human lymphocyte populations, primarily EBV's main host cell, the human B cell. In this protocol we describe human immune system reconstitution after neonatal transfer of CD34^+^ hematopoietic progenitor cells in lymphodeplete immune compromised mouse strains, using NOD‐*scid* γ_c_^–/–^ (NSG) mice as a commonly used example. Such reconstituted humanized mice allow intraperitoneal and intranasal infection with EBV and we describe injection of 10^5^ infectious particles of the prototypic EBV strain B95‐8 that can be produced from a recombinant bacmid (p2089) in HEK293 cells. Infection with this dose mimics symptomatic primary EBV infection, infectious mononucleosis (IM), with high viral loads plateauing 4 weeks after infection, with CD8^+^ T‐cell lymphocytosis at week 5 and 6 after infection. This IM‐like primary EBV infection in humanized mice leads to clonal EBV‐induced B‐cell lymphoproliferations that resemble large B‐cell lymphomas with the latency III program of EBV infection. We describe Basic Protocols to monitor viral loads, immunohistochemistry of infected tissues and spectral flow cytometry to characterize protective T‐cell expansion. The described mouse model has been used by us and others to characterize mutant EBV infections, cell‐mediated immune control of EBV, modulation of EBV pathogenesis by co‐infections with human immunodeficiency virus (HIV) and Kaposi sarcoma associated herpesvirus (KSHV), as well as passive transfer of vaccine elicited antibodies to test their protection against EBV infection. © 2025 The Author(s). Current Protocols published by Wiley Periodicals LLC.

**Basic Protocol 1**: CD34^+^ human hematopoietic progenitor cell isolation and characterization

**Basic Protocol 2**: Human immune system reconstitution and characterization

**Basic Protocol 3**: Recombinant EBV production and humanized mouse infection

**Basic Protocol 4**: Viral load quantification, lymphoma assessment, and immunohistochemistry after EBV infection of humanized mice

**Basic Protocol 5**: Human T‐cell response analysis after EBV infection of humanized mice

## INTRODUCTION

Mice with reconstituted human immune systems (humanized mice) have in part been developed to study human lymphotropic pathogens, initially primarily for human immunodeficiency virus (HIV) research (McCune et al., 1988; Stripecke et al., 2020). Instead of human peripheral blood mononuclear cell (PBMC) transfer, humanized mice allow for long term hematopoietic multilineage reconstitution without rapid graft‐vs‐host‐disease (GvHD) that originates from mature T‐cell transfer with PBMCs due to their education outside the mouse host. Indeed such a long reconstitution is required for infection with the B‐cell tropic Epstein Barr virus (EBV), whose viral loads only peak 4 to 6 weeks after infection, as observed in symptomatic primary EBV infection called infectious mononucleosis (IM) (Damania et al., 2022; Farrell, 2019; Münz, 2025; Taylor et al., 2015). We and others have observed that CD8^+^ T cells expand and CD4^+^ T cells become activated in response to EBV infection in humanized mice, that these T cells recognize autologous EBV transformed B‐cell lines, and that T‐cell depletion elevates viral loads and increases EBV associated large B‐cell lymphoma formation (Melkus et al., 2006; Shultz et al., 2010; Strowig et al., 2009; Traggiai et al., 2004). Since then, humanized mice have been used to characterize phenotype, co‐stimulatory requirements, and antigen specificities of EBV‐specific CD8^+^ T‐cell responses (Antsiferova et al., 2014; Caduff et al., 2020; Chatterjee et al., 2019; Chijioke et al., 2015; Deng et al., 2021; Kirchmeier et al., 2024; Volk et al., 2020). Innate immune responses to EBV, including by natural killer (NK) and by dendritic cells (DCs), have been investigated in humanized mice (Caduff et al., 2021; Chijioke et al., 2013; Gujer et al., 2019; Landtwing et al., 2016). Furthermore, mutant EBV viruses and different viral isolates have been characterized in humanized mice (Ma et al., 2011, 2015; Murer et al., 2018, 2019; Tsai et al., 2013, 2017; White et al., 2012). The modulation of EBV‐associated pathogenesis by co‐infections, including HIV and Kaposi's sarcoma‐associated herpesvirus (KSHV), have been studied (Caduff et al., 2024; McHugh et al., 2017, 2020). Finally, humanized mice have even been used to shed some light on the connection between EBV infection and autoimmune diseases, primarily multiple sclerosis (MS) (Laderach et al., 2025; Zdimerova et al., 2021). While humanized mice have proven to be a versatile tool to study EBV infection, associated lymphomagenesis, and cell‐mediated immune control, they are poorly suited to investigate EBV‐specific antibody responses, epithelial cell infection by EBV, and assess EBV associated pathologies beyond latency III EBV gene products expressing large B‐cell lymphomas or primary effusion lymphomas (PELs), the latter due to co‐infection with KSHV. Here, we describe the most used humanized mouse model for EBV infection. Therefore, we will outline protocols for CD34^+^ human hematopoietic progenitor cell isolation and characterization, human immune system reconstitution in immune compromised mice, recombinant EBV production and humanized mouse infection, viral load and EBV‐associated lymphoma characterization, and basic T‐cell characterization in response to EBV infection.

## STRATEGIC PLANNING

**Figure 1:**
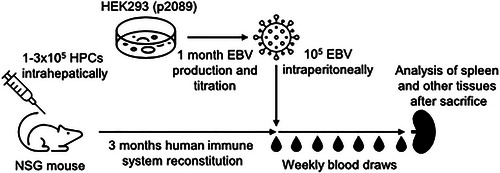
Schematic depiction of humanized mouse reconstitution and EBV infection.

Performing EBV infection in humanized mice requires a significant amount of planning, starting from securing CD34^+^ human hematopoietic progenitor cells (HPCs) and the respective immune‐compromised mouse strains. Breeding pairs from the respective mouse strains need to be established for neonatal injection of HPCs. The reconstitution of human immune systems then takes 3 months (Fig. 1). In parallel, recombinant EBV needs to be produced and the infectious titer determined, which also often takes 1 month. Finally, the EBV infection of humanized mice is carried out for 4 to 6 weeks, during which time the infected mice must be closely monitored for declining health. The infection and cell‐mediated immune response can be longitudinally monitored in weekly blood draws.

*CAUTION*: The Epstein Barr virus (EBV) is a Biosafety Level 2 (BSL‐2) pathogen. Follow all appropriate guidelines and regulations for the use and handling of pathogenic microorganisms.

*NOTE*: All protocols involving animals must be reviewed and approved by the appropriate Animal Care and Use Committee and must follow regulations for the care and use of laboratory animals. Appropriate informed consent is necessary for obtaining and use of human study material.

## Basic Protocol 1 CD34^+^ HUMAN HEMATOPOIETIC PROGENITOR CELL ISOLATION AND CHARACTERIZATION

This protocol outlines the isolation of CD34^+^ human hematopoietic progenitor cells from human fetal liver tissue. First, the liver tissue is digested and mashed into a suspension from which mononuclear cells (MCs) are isolated by density gradient centrifugation using Ficoll‐Paque. Following isolation, cells are labeled with CD34‐specific magnetic beads and subjected to magnetic‐activated cell sorting (MACS). To maximize purity, the sorting process is performed twice using two consecutive MACS columns. The success of the enrichment is assessed by flow cytometry. The resulting CD34^+^ cells are then cryopreserved until they are needed for human immune system reconstitution in newborn mouse pups.

### Materials

Human fetal liver tissue (procured by Cercle Allocation Services)
Digestion mix (see recipe)
Sanosil S010 (Sanosil AG, 12010005CH)
0.5 M EDTA, pH 8.0 for molecular biology (BioFroxx, 1353ML500)
RPMI (Gibco, 7001612)
Ficoll‐Paque (GE Healthcare, 17‐5442‐03)
Trypan‐blue solution, 0.4% (Gibco, 15250061)
Magnetic activated cell sorting (MACS) buffer (see recipe)
Direct CD34 progenitor cell isolation kit, human (Milteny, 130‐046‐703)
Dimethyl sulfoxide (DMSO) (Merck, S0615‐500ML)
Fetal calf serum (FCS) (Sigma‐Aldrich, D8418)
Phosphate‐buffered saline (PBS) (in house; see Current Protocols, 2006)
Anti‐human CD34‐APC (invitrogen, CD3458105)
Anti‐human CD38‐PE (Biolegend, 303506)
Zombie Aqua fixable viability kit (Biolegend, 423101)
Paraformaldehyde solution, 4% in PBS (ChemCruz, sc‐281692), optional

Herasafe laminar flow hood; class II (Thermo Fisher Scientific)
15‐ and 50‐ml plastic Falcon tubes (Sarstedt, 7510521 and 7510105)
10‐cm Corning Petri dishes (Corning, 7000513)
Surgical scissors (autoclaved)
Surgical tweezers (autoclaved)
Parafilm (Pechiney Plastic Packaging)
Rocking shaker (Faust)
Cell strainer, 70‐µm (Falcon, 7002108)
2‐ml syringes, Injekt Luer Solo (B. Braun Medical AG, 4606027V)
Pipetgirl (Vitaris)
10‐, 20‐, 100‐, 200‐, and 1000‐µl pipettes and tips (Eppendorf)
Centrifuge, Sorvall ST40R (Thermo Fisher Scientific)
Transfer pipette 3.5 ml (Sarstedt, 86.1171.001)
5‐ml polystyrene round‐bottom tube (Falcon, 352235)
Ice bucket with ice (Corning)
Counting chamber, BLAUBRAND (Brand, 717805)
MACS‐Mix tube rotor (Miltenyi)
MACS MultiStand (Miltenyi)
MidiMACS magnet (Miltenyi)
LS columns (Miltenyi, 130‐042‐401)
Mr. Frosty freezing container (Milian, 5100‐0001)
TSX Universal Series general purpose ultra‐low freezer (Thermo Fisher Scientific)
Liquid nitrogen tank (MVE Cryosystem 4000)
BD FACSCanto flow cytometry system
FlowJo software v10.10.0 (BD Life Sciences)

#### Tissue digestion

All steps should be performed in a BSL‐2 laboratory in an appropriate laminar flow hood (tissue is tested for HIV, hepatitis B (HBV), and hepatitis C (HCV) viruses; but the test results arrive later than the shipment).

1 Decant the shipping medium into a new 50‐ml Falcon tube. Transfer the liver and smaller tissue pieces into a 10‐cm Petri dish.
2 Check the tissue thoroughly for a gallbladder. If the gallbladder is still attached, remove it carefully without causing damage to protect the tissue from the digestive fluids.
3 Use the autoclaved surgical scissors and tweezers to remove the white connective tissue and fat. Then, cut the liver into very small pieces in the Petri dish.
  Tip: You can place the connective tissue and fat on the Petri dish lid.
4 Transfer the tissue mix back to the 50‐ml tube it was received in. Add half of the digestion mix to the Petri dish to facilitate the transfer of remaining tissue pieces. Use the other half of the digestion mix to rinse the scissors and tweezers, collecting all liquid in the 50‐ml tube.
5 Cover the tube with Parafilm, put it in a shaker, and incubate at room temperature for exactly 25 min.
  Decontaminate the scissors and tweezers in Sanosil S010 for 30 min before rinsing both with water and autoclaving.
6 Add EDTA to the 50‐ml tube at a final concentration of 10 mM (dilute 1:50, i.e., 200 µl per 10 ml) and shake the tube again for 5 min at room temperature.
7 Fill the tube with RPMI to quench the digestion.
8 Mash and filter the tissue mix through a 70‐µm cell strainer into a new 50‐ml tube, using the pestle of a 2‐ml syringe to mash the suspension. Use RPMI to wash the cell strainer repeatedly.
  Start with the larger tissue pieces at the bottom of the tube. Change the filter when the bottom is covered with connective tissue. Every time you change the filter, remove the liquid from the bottom of the cell strainer with a 1‐ml pipette and add the liquid to the cell suspension.

#### Mononuclear cell isolation by gradient centrifugation

9 Prepare two 50‐ml tubes with 15 ml Ficoll‐Paque. Carefully, layer 20 to 35 ml of cell suspension onto the 15 ml Ficoll‐Paque with a Pipetgirl. Tilt the tube and pipette very slowly to not disturb the two forming layers.
10 Centrifuge 30 min at 1000 × *g*, room temperature. Use an acceleration of 1 and a deceleration of 3 to preserve the distinct layers during centrifugation.
11 After centrifugation, the following layers will be visible from top to bottom: plasma (cloudy, yellow), mononuclear cells (MCs, thin white/gray band), Ficoll‐Paque (clear), red blood cells (dark red pellet). Use a transfer pipette to carefully transfer the MC layer to a new 50‐ml tube. Merge the MCs from both Ficoll‐Paque tubes into one new 50‐ml tube.
  Make sure to collect the whole cell layer. Transferring serum or Ficoll‐Paque with the PBMC layer is not a problem and will be removed by the washing step afterwards.
12 Fill the tube to the top with RPMI and centrifuge 10 min at 500 × *g*, 4°C.
13 Resuspend the cell pellet in 5 ml RPMI and filter it again through a 70‐µm cell strainer into a new 50‐ml tube.
  Wash the old tube with 5 ml RPMI and transfer it to the cell strainer as well.
14 Fill the tube to 50 ml with RPMI. Remove 50 µl of cell suspension for flow cytometry staining and transfer it to a 5‐ml polystyrene tube. Label the tube with “before MACS”. Store the tube on ice.
15 Remove 10 µl of the cell suspension for counting and dilute it with trypan blue (∼1:20). Use 10 µl of the dilution to count the cells using a counting chamber.
16 Centrifuge the rest of the cell suspension 10 min at 500 × *g*, 4°C.

#### MACS labeling and separation

17 Resuspend the pellet in 600 µl MACS buffer per 10^8^ cells.
  It is vital that the MACS buffer is kept at 4°C or on ice for the whole MACS separation to reduce unspecific binding and ensure optimal experimental conditions.
18 Filter the cell suspension through a 70‐µm cell strainer into a new 15‐ml tube.
  Tip: Invert the cell strainer on the tube. If you have >5 ml cell suspension, use a 50‐ml tube.
19 Add 100 µl FcR blocking reagent per 10^8^ cells from the CD34 progenitor cell isolation kit and incubate the cell suspension on ice for 5 min. Mix well before incubation.
20 Add 100 µl CD34 beads per 10^8^ cells to the cell suspension and incubate for 30 min at 4°C on the MACS‐Mix rotor.
21 Wash cells with 10 ml MACS buffer and centrifuge 10 min at 500 × *g*, 4°C.
22 Resuspend cells in 1.5 ml per 10^8^ cells in MACS buffer. Use a minimum of 3 ml.
23 Filter the cell suspension through a 70‐µm cell strainer into a new 15‐ml tube.
24 Prepare the MACS equipment:
  a. Place the metal holder in the hood and install the magnets.
  b. Affix the LS column to the magnet.
  c. Equilibrate the column by rinsing it with 5 ml MACS buffer.
  d. Place an empty 50‐ml tube underneath the column to collect all flow‐through.
    For all following steps, it is important to wait until all liquid has passed before continuing with the next step. Make sure a new drop is not forming before adding more liquid to the top of the column.
25 Add the cell suspension and collect the flow‐through. Label the tube with “CD34‐negative fraction”.
26 Wash the column three times with 3 ml MACS buffer.
27 Remove the column from the magnet and place it on a new 15‐ml tube. Add 5 ml MACS buffer and flush out all positive cells using the pestle that belongs to the column.
28 Repeat the MACS separation a second time by placing a new column on the magnet (repeat steps 24 to 27).
29 Remove 50 µl from the negative fraction and add it to a polystyrene tube. Label the tube with “CD34neg”.
30 Remove 50 µl from the positive fraction and add it to a polystyrene tube. Label the tube with “CD34pos”.
31 Remove another 50 µl from the positive fraction and add it to another polystyrene tube. Label the tube with “unstained”. Store all polystyrene tubes on ice.

#### Freezing of isolated cells

32 Calculate how many tubes of the positive and negative fraction will be frozen down: freeze 2 × 10^6^ CD34^+^ cells per tube and distribute the CD34^–^ cells equally to four tubes with maximally 10^8^ per cryovial.
  The CD34^–^ cells are useful for both B‐cell isolation and autologous EBV transformed B‐cell (LCL) generation, as well as HLA typing.
33 Prepare 1 ml of freezing medium per tube: supplement RPMI with 7.5% DMSO and 20% FCS.
34 Centrifuge the cell suspension 10 min at 500 × *g*, 4°C. Resuspend the pellet in the appropriate volume of freezing medium and distribute 1 ml of cell suspension to each freezing tube. Label the tubes thoroughly.
35 Place the tubes in a Mr. Frosty and transfer it to –80°C.
36 Transfer the tubes to a liquid nitrogen tank the next day. Thaw only when newborn pups are available for intrahepatic injection (see Basic Protocol 2).

#### Flow cytometry staining, acquisition, and analysis

**Figure 2:**
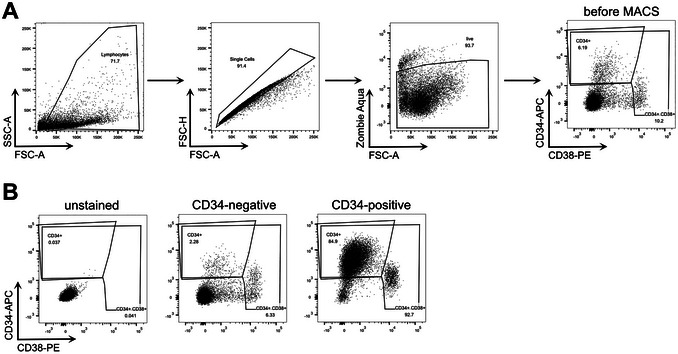
Flow cytometry characterization of hematopoietic progenitor cells, isolated from human fetal liver tissue. (**A**) Gating strategy for the identification of hematopoietic progenitor cells, defining the lymphocytic target population and excluding duplets as well as dead cells. Progenitor cells are then characterized by CD34 and CD38 expression. (**B**) Example flow cytometry plots showing the enrichment of CD34^+^ cells after MACS, compared to CD34‐negative fraction and before MACS. The unstained control sample or the “before MACS” sample can be used to set the gates for CD34‐APC and CD38‐PE.

37 Prepare the staining mix in PBS. Calculate a total volume of 30 ml per sample. The appropriate dilutions are as follows:
  a. CD34‐APC, 1:50.
  b. CD38‐PE, 1:100.
  c. Zombie‐Aqua, 1:500.
38 Add 30 µl staining mix per sample (do not stain the “unstained” sample) and incubate at 4°C for 30 min in the dark.
39 Wash the samples by adding 2 ml PBS and centrifuge 5 min at 500 × *g*, 4°C. Resuspend the pellet in 100 µl PBS.
  If the samples cannot be acquired the same day, the samples can be fixed with 1% PFA for 15 min.
40 Acquire the samples on a BD FACSCanto flow cytometry system.
41 Upload all sample files to a FlowJo workspace.
42 Follow the gating strategy as shown in Figure 2A.
  a. The first gate defines the target cell population (lymphocytes), the second gate excludes duplets, and the third gate excludes dead cells.
  b. CD34 and CD38 are then used as markers to identify hematopoietic stem cells (CD34^+^/CD38^–^) and already further differentiated progenitor cells (CD38^+^).
  c. Use the unstained sample to define the gates.

43 Validate the success of the MACS and the quality of the isolated CD34^+^ cell population by comparing all stained samples (Fig. 2B). Record the frequency of CD34^+^ cells for further calculations in Basic Protocol 2.
  Additionally, recording the frequency of live cells can help to evaluate the cell quality before reconstitution in Basic Protocol 2.

## Basic Protocol 2 HUMAN IMMUNE SYSTEM RECONSTITUTION AND CHARACTERIZATION

This protocol outlines the reconstitution and characterization of NSG mice with hematopoietic progenitor cells (HPCs) derived from human fetal liver tissue. Newborn NSG mouse pups bred in‐house will be irradiated with 1 Gray. After ∼5 hr, the isolated CD34^+^ HPCs will be thawed and counted. 5 to 7 hr after irradiation, CD34^+^ HPCs will be injected into the pups’ livers. Characterization of NSG mice will be performed 12 weeks after reconstitution. The mice will be bled from the tail vein. The blood will be lysed and stained for flow cytometry acquisition. The success of the human immune system engraftment is assessed using flow cytometry.

### Materials

NSG mice: NOD.Cg‐*Prkdc^scid^ Il2rg^tm1Wjl^*/SzJ (Jackson, strain no. 005557, RRID:IMSR_JAX:005557)
Virkon S (Lanxess)
CD34^+^ cells (see Basic Protocol 1)
PBS, pH 7.4, sterile (Gibco,10010‐023)
70% ethanol (VWR, 20821.321)
Trypan blue solution, 0.4% (Gibco, 15250061)
ddH_2_O (Millipore filter system, in house)
Heparin‐Na 25.000 I.E./5 ml (Braun, EAN:4150157826988)
1× ACK lysis buffer (see recipe)
Antibodies:
  Anti‐human CD45‐PB (Biolegend, 304029)
  Anti‐human CD3‐PE (Biolegend, 300408)
  Anti‐human CD4‐APC‐Cy7 (Biolegend, 300518)
  Anti‐human CD8‐PerCP (Biolegend, 344708)
  Anti‐human CD19‐PE‐Cy7 (Biolegend, 302216)
  Anti‐human HLA‐DR‐FITC (Biolegend, 307604)
  Anti‐human NKp46‐APC (BD, 558051)
1% PFA (ChemCruz, sc‐281692)
BD CompBeads, Anti‐Mouse Ig, κ/Negative Control, FBS (BD Pharmingen, 552843)

Hamilton 100‐µl syringe, 1710 TLL no STOP P/N: 8120/01, autoclaved (Thermo, 10267820)
Hamilton NDL N 6/pk ga33/13 mm/pst4‐12°, autoclaved (BGB Analytik AG, HA‐7747‐01‐16)
Irradiation cage (in house)
Cosmetic paper tissue, autoclaved (Weita AG, 2015.10018)
Sterile cotton pads (Roth, NK84.1)
Herasafe laminar flow hood; class II (Thermo Fisher Scientific)
RS 2000 X‐ray Biological irradiator (Rad Sources)
Colored tape, multiple colors (Thermo Fisher, 15824565)
V‐bottom plastic tubes (Sterilin, Thermo Fischer Scientific, 11339133)
Water bath, 36°C
10‐, 20‐, 100‐, 200‐, and 1000‐µl pipettes and tips (Eppendorf)
Pipetgirl (Vitaris)
5‐, 10‐, and 25‐ml serological pipettes (Sarstedt)
Centrifuge, Sorvall ST40R (Thermo Fisher Scientific)
Counting chamber, BLAUBRAND (Brand, 717805)
1.5‐ml SafeSeal tubes (Sarstedt, 72.706)
Redlight construction (in house)
Scalpel blades, size 10 (Aesculp, BB510)
FACS tubes
Vortex mixer
BD FACSCanto flow cytometry system
FlowJo software v10.10.0 (BD Life Sciences)

*CAUTION*: The needles can easily bend and become unusable.

#### Preparation

1 To reconstitute an appropriate number of NSG pups, establish an in‐house breeding system with 6 to 8 NSG breeders.
2 Autoclave Hamilton needles and syringes, irradiation cage, cosmetic paper tissue, and cotton pads.
3 Use Virkon to wipe down all surfaces that could come into contact with mice for at least 5 min.

#### Irradiation of newborn mouse pups

All steps should be performed in an appropriate laminar flow hood.

4 Mouse pups should be irradiated on day 1 (only if the milk spot is visible and the female breeder is not within the process of giving birth anymore) to day 5; up to day 7 is possible.
5 Prepare two sterile cosmetic paper tissues next to the irradiation cage.
  Place the first tissue paper between the breeder's cage and the irradiation cage. Place a second tissue on top of the first to create a sterile environment for the pups.
6 Carefully remove pups from the parents’ cage by cupping them with nesting material and transfer them to the prepared tissues, ideally without touching the pups.
7 Roll the sterilized paper towels over the pups and make sure that they stay together inside the paper towels.
  Use colored tape to mark both the parent's cage and the correct paper tissue holding the related litter. You can place several litters in one irradiation cage. Use fresh, sterilized paper tissue for each litter.
8 Close the irradiation cage and bring it to the irradiation unit.
9 Mouse pups of radiosensitive mouse strains carrying the *Prkcd^scid^* mutation will be irradiated with 1 Gray. Note the irradiation time.
10 Bring the pups back to the respective mouse facility under the laminar flow and transfer the pups back to their parents’ cage, ideally without touching the pups. Monitor if the mother accepts the pups.

#### Reconstitution

11 Reconstitute the pups 5 to 7 hr after irradiation.
  All animals reconstituted from the same CD34^+^ donor belong to one cohort. One cohort should comprise 15 to 30 mice to set up an experiment. Litters of different breeder cages (from the same strain) can be reconstituted with cells from the same donor. One donor can be used for several reconstitutions to achieve sufficient animal numbers for one cohort.
12 Each pup will be injected with 200,000 CD34^+^/CD38^–^ cells.
  Due to injection errors, cells should be prepared for 1 or 2 additional pups (e.g., 10 pups to inject → 12 pups × 0.2 × 10⁶ = 2.4 × 10⁶ cells/pup). See Table 1 for calculation examples. Check the purity of the CD34^+^/CD3^–^ cell fraction using Basic Protocol 1. If the purity of the population is <90%, adjust the total cell numbers for injection accordingly.

**Table 1:** Exemplary Calculation for the Preparation of CD34^+^CD38^–^ Cells for Injection

No. of pups	Cell no. needed for injection (CD34^+^/CD38^–^)	Calculation (including 2× for injection errors and variability of recovery ∼70%‐100%)	Minimum no. of total cells needed (purity of CD34^+^ >90%)	No. of vials (∼2 × 10^6^ cells per vial)	More reconstitutions needed with this donor?
5	1.0 × 10^6^	(5 pups × 2 × 10^5^ cells) + (2 × 2 × 10^5^) = 1.4 × 10^6^ cells	∼ 1.5–2.0 × 10^6^	1	Yes
8	1.6 × 10^6^	(8 pups × 2 × 10^5^ cells) + (2 × 2 × 10^5^) = 2.0 × 10^6^ cells	∼ 2.5–3.0 × 10^6^	1‐2	Yes
10	2.0 × 10^6^	(10 pups × 2 × 10^5^) + (2 × 2 × 10^5^) = 2.4 × 10^6^ cells	∼ 3.0–3.5 × 10^6^	2	Yes
15	3.0 × 10^6^	(15 pups × 2 × 10^5^) + (2 × 2 × 10^5^) = 3.4 × 10^6^ cells	∼ 4.0–4.5 × 10^6^	2‐3	Yes, if more cells are available
20	4.0 × 10^6^	(20 pups × 2 × 10^5^) + (2 × 2 × 10^5^) = 4.4 × 10^6^ cells	∼ 5.0–5.5 × 10^6^	3‐4	Yes, if more cells are available

13 Prepare 2 ml PBS (per cryotube you want to thaw) in one V‐bottom plastic tube. Do not pool >3 cryovials in one V‐bottom tube.
14 Retrieve the cells from the liquid nitrogen.
15 Thaw the vial in a water bath. As soon as only a small piece of ice is left (to keep it at 4**°**C), clean the vial with ethanol and transfer it to the hood.
16 Use a 1000‐µl pipette with a filter tip to transfer the cells to the plastic tube.
17 Remove 10 µl for counting and another 10 µl as a backup for counting.
18 Fill the plastic tube with PBS using a Pipetgirl with serological pipettes and centrifuge 10 min at 400 × *g*, 4°C.
19 Count the cells in the meantime using trypan blue and the counting chamber.
20 Calculate the cell number and check if the cell number is sufficient to inject all pups planned. An exemplary calculation can be found in Table 1.
  If you do not have enough cells for all pups, you can either prepare more cells from the same donor or, if this is not possible, excess pups must be euthanized.
  If it is necessary to decrease the number of pups, please avoid selecting all pups from a single litter for euthanasia. Doing so could impact the parents’ behavior and potentially lead to issues with the next litter.
21 Resuspend the cells in the correct volume of PBS (20 µl/mouse + 2× for injection error, e.g., 10 pups + 2 pups → 240 µl)
  Please work very precisely at this step. There may be residual liquid left in the plastic tube from the aspiration, which could influence the actual cell concentration vs the one calculated for one injection.

#### Intrahepatic injection of cells

22 Prepare one Hamilton syringe for each donor by carefully screwing the needle onto the syringe. Use sterile PBS to moisten the syringe before you start.
  The needles can easily bend and become unusable. You can use the same needle for all pups receiving cells from the same donor. Only use a new needle if the old one is blunt or broken.
23 Always wet gloved hands with Virkon and spread over both hands before touching the animals.
24 Gently pick up the mouse pup by its neck. Hold the pup upside down with its head facing towards your hand and its belly up. Inject each pup with 20 µl of cell suspension intrahepatically on the right side of the abdominal cavity (left side when the mouse abdomen is facing you).
25 Gently put the pups back in their cage. Monitor if the mother accepts the pups before transferring the breeder cage to the rack.
26 Dispose the needle after all pups have been injected with cells from this donor.
  Needles are single use only; the syringe should be flushed with ddH_2_O and ethanol, dried and prepared for autoclaving.
27 Reconstitution efficacy should be determined 12 weeks after injection.

#### Blood collection 12 weeks after reconstitution

All steps should be performed in an appropriate laminar flow hood.

**Figure 3:**
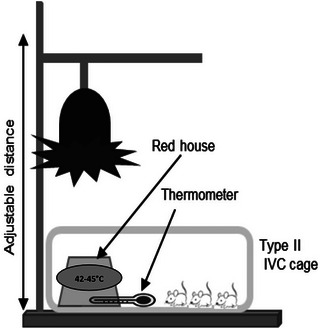
Build‐up scheme of IR lamp apparatus. Infrared (IR)‐lamp is fixed with a stand (lamp holder) that enables adjusting the distance between lamp and cage. Cage is placed under the lamp so that less than half the cage is directly expose to the light. A red animal house is placed under the lamp in the center of the light cone. A thermometer is placed with its sensor inside the light cone.

28 Prepare 20 µl heparin per Eppendorf tube and label it with the animal number.
29 Place the ACK lysis buffer at room temperature.
30 Disinfect the infrared (IR)‐lamp apparatus with 70% ethanol and transfer the lamp into the laminar flow hood.
31 Place a cage with bedding and a red house under the lamp. Arrange the cage so that less than half of the cage is directly exposed to the light and the lamp 5 to 10 cm from the top of the cage. Place the red house in the illuminated area, directly under the light bulb. Place the thermometer in the light cone (Fig. 3).

32 Take up to 5 animals from their home cage and place them in the heating cage. Optional: Place the grid on top of the cage if wanted.
33 Switch on the IR lamp to warm mice.
34 Keep the temperature between 42° and 45°C.
  Monitor mice for signs of hyperthermia or overheating. If overheating is observed, remove the affected animal from the cage and process it for subsequent procedure, e.g., i.v. injection or blood sampling (see “Potential adverse effects” at the end of this protocol).
35 Collect 80 µl of venous blood per animal by carefully cutting the tail vein. Vortex the blood right away to avoid clumping.
36 Label FACS tubes and add 1 ml of red blood cell lysis buffer (i.e., ACK lysis buffer) per tube.
37 Add 500 µl of red blood cell lysis buffer directly to the Eppendorf tubes containing the blood.
38 Transfer blood plus lysis buffer from the Eppendorf tubes into the FACS tubes
39 Vortex and incubate for exactly 5 min at room temperature.
40 Wash with cold PBS by adding PBS to the top of the tube.
41 Centrifuge 5 min at 500 × *g*, 4**°**C.
42 Decant the supernatant and remove last drops by dipping on a paper tissue.
43 Add 500 µl lysis buffer and resuspend using a 1000‐µl pipette.
44 Incubate for exactly 5 min at room temperature.
45 Wash with cold PBS by adding PBS to the top of the tube.
46 Centrifuge 5 min at 500 × *g*, 4**°**C.
47 Decant the supernatant and remove last drops by dipping on a paper tissue.
48 Check if most of the red blood cells have been lysed. If not, repeat lysis once again.

#### Flow cytometry staining, compensation, acquisition, and analysis

49 Prepare the staining mix in PBS. Calculate a total volume of 30 µl per sample. The appropriate dilutions are as follows:
  a. CD45‐PE, 1:400.
  b. HLA‐DR – FITC, 1:200.
  c. All other antibodies, 1:100.
50 Add 30 µl antibody mix per sample and resuspend well using a pipette and vortex.
51 Incubate 30 min at 4**°**C in the dark.
52 Wash with 2 ml PBS.
53 Centrifuge 5 min at 500 × *g*, 4**°**C.
54 Resuspend in 100 µl PBS if you acquire the samples right away. Skip steps 55 and 56.
55 Resuspend in 100 µl 1% PFA if you acquire >2 hr later or the next day.
56 Incubate for 30 min at 4°C, wash with 2 ml PBS, centrifuge 5 min at 500 × *g*, 4**°**C, resuspend in 100 µl PBS, and store at 4°C.
57 Mix both reagents of BD CompBeads (1:1, e.g., by adding 1 drop of each bottle; 1 drop = 25 to 30 µl).
58 Prepare one FACS tube for each single stain and one for an unstained control.
59 Add 15µl CompBead mix to the single stain tube.
60 Add <0.4 µl of antibody and vortex.
61 Incubate 15 min in the dark at 4**°**C.
62 Add 100 µl PBS.
63 Acquire single stains and samples on a BD FACSCanto flow cytometry system. Link and save the compensations before acquiring the samples.
  The CompBeads are much smaller than the cells. Adjust the FSC accordingly.
64 Upload all sample files to a FlowJo workspace.
65 Follow the gating strategy shown in Figure 2.

The first gate is a time gate for identification and removal of artifacts or events that occur due to issues like air bubbles, clogs, or unstable fluidics. The second gate defines the target cell population (leucocytes and lymphocytes), the third gate excludes duplicates, and the fourth gate excludes dead cells. CD45 is used as a marker to identify common human leukocytes. The gates for CD3 and CD19 identify T and B cells, respectively. Within the CD3 population, the CD4 and CD8 markers identify T helper cells and cytotoxic T cells, respectively. HLA‐DR is an activation marker used for T cells and NKp46 identifies the NK cells.

#### Potential adverse effects

Overheating of mice must be avoided under any circumstances as it may influence the animal well‐being and health. An early sign of overheating of a mouse can be that it buries itself in the cage bedding. A late sign of overheating can be inactivity or when the mouse is lying belly down with outstretched limbs.

## Basic Protocol 3 RECOMBINANT EBV PRODUCTION AND HUMANIZED MOUSE INFECTION

This protocol outlines a standardized method for the production and quantification of recombinant EBV from a HEK293‐based producer cell line (p2089 cells) (Delecluse et al., 1998, 1999), harboring the EBV genome and a GFP reporter, followed by intraperitoneal (i.p.) injection into humanized NSG mice for in vivo studies. The EBV lytic cycle is induced by transfecting p2089 cells with BZLF1 and BALF4 expression plasmids, leading to the release of infectious virus into the culture supernatant. Subsequently, the viral titer is determined by infecting Raji cells and quantifying GFP expression using flow cytometry. Based on these measurements, the desired infectious dose is calculated and administered to humanized mice.

This protocol includes three main parts: (1) production and concentration of EBV in producer cells; (2) quantification of viral infectivity by Raji cell titration and flow cytometric analysis; and (3) i.p. injection of the virus into humanized mice.

### Materials

p2089 cells [human embryonic kidney 293 (HEK293) cells, carrying the GFP gene and harboring the recombinant EBV strain B95‐8 DNA as a bacmid (p2089) under hygromycin selection (Hammerschmidt lab; Helmholtz Center Munich, Germany)]
R10 medium (see recipe)
Hygromycin B, 50 mg/ml (Invitrogen, 10687010)
Trypsin‐EDTA, 0.05% (Gibco, 25300054)
Trypan blue solution, 0.4% (Gibco, 15250061)
Opti‐MEM (Gibco, 31985054)
p509 BZLF1 expression vector (Hammerschmidt & Sugden, 1988)
p2670 BALF4 expression vector (Neuhierl et al., 2002)
PEI MAX, 1 mg/ml (Polysciences, 24765‐100)
PBS (Gibco, 10010023)
Sanosil S010 (Sanosil, 12F10005CH)
Raji cells (ATCC, CCL‐86)
Zombie Aqua fixable viability kit (Biolegend, 423102)
Virkon S (Lanxess)
NSG mice: NOD.Cg‐*Prkdc^scid^ Il2rg^tm1Wjl^*/SzJ (Jackson, strain no. 005557, RRID:IMSR_JAX:005557)
Humanized NSG mice: NSG mice reconstituted with CD34^+^ human hematopoietic progenitor cells (see Basic Protocol 2)
Ethanol (VWR, 20821.321)

Herasafe laminar flow hood; class II (Thermo Fisher Scientific)
Liquid nitrogen tank (Thermo Fisher Scientific, CY509108)
15‐cm cell culture dishes (Corning, 353025)
Centrifuge, Sorvall ST40R (Thermo Fisher Scientific)
Incubator (Binder)
Counting chamber, BLAUBRAND (Brand, 717805)
10‐, 20‐, 100‐, 200‐, and 1000‐µl pipettes and filter tips (Eppendorf)
Vortex mixer
1.2‐µm syringe filters (Sigma Aldrich, WHA10462260)
Centrifuge tubes, polycarbonate (Thermo Fisher Scientific, 3138‐0050)
Sorvall RC 6+ centrifuge (Thermo Fisher Scientific, 46915)
SS34 centrifuge rotor (Thermo Fisher Scientific, 28020)
Scale (Kern, TADJ 200‐4‐A)
Aspiration pipettes (Sarstedt, 86.1252.011)
Vacuum pump (Integra Biosciences)
1.5‐ml SafeSeal tubes (Sarstedt, 72.706)
Flat‐bottom 96‐well cell culture plate (Corning, 353072)
FACS tubes with cell‐strainer caps (Corning, 352235)
BD FACSCanto II flow cytometer
FlowJo software v10.10.0 (BD Life Sciences)
Nitrile gloves (Sempermed)
0.5‐ml insulin syringes (BD, 324824)
Ice bucket with ice (Corning)
Additional basic equipment:
  Pipetgirl (Vitaris)
  Serological pipettes (Sarstedt, 86.1254.001)
  Plastic tubes, 50‐ and 15‐ml (Sarstedt, 7510521 and 7510105)
  50‐ml syringes, Omnifix Luer Lock Solo (B. Braun, 4617509F)

#### Virus production

All steps should be performed in a BSL‐2 laboratory in an appropriate laminar flow hood.

1 Thaw p2089 cells from liquid nitrogen storage, wash with R10 medium, and use the Sorvall ST40R centrifuge to spin cells down for 5 min at 500 × *g*, 4°C. Resuspend the pellet in R10 to plate the cells into 15‐cm dishes. Culture them at 37°C with 5% CO_2_.
2 After the first splitting, culture cells in R10 medium supplemented with 100 µg/ml hygromycin and split at 70% to 80% confluency using trypsin.
  Hygromycin must be added after the first splitting to prevent loss of the EBV bacmid.
3 The day before transfection, seed 5 million p2089 cells in 15‐cm dishes after cell number determination with a counting chamber and trypan blue solution.
4 If the cells have a confluency of ∼70% on the next day, change the medium to R10 without hygromycin supplementation and prepare the transfection mix:
  a. Per 15‐cm dish, prepare 3 ml Opti‐MEM and add 3 µg of each p509 and p2670.
  b. Mix well.
  c. Add 36 µl PEI MAX (1 mg/ml) dropwise.
  d. Vortex and incubate at room temperature for 15 to 20 min.
  e. Add dropwise to the cells and incubate at 37°C.
5 Three days after transfection, collect the supernatant and spin it down for 10 min at 2000 × *g*, 4°C, to remove the cellular debris. Filter through a 1.2‐µm filter and store at 4°C for up to 1 week or proceed directly to the next step.
6 Centrifuge the supernatant to concentrate the virus as follows:
  a. Fill the virus supernatant in centrifugation tubes and, using a Sorvall RC 6+ centrifuge with SS34 rotor, spin down for 2 hr at 14,000 × *g*, 4°C.
    Weigh the centrifugation tubes with a scale before starting the run, since the centrifuge needs to be balanced out exactly.
    Mark the side where you expect your virus to be after centrifugation; the pellet will be invisible to the eye.
  b. Aspirate the supernatant, add 100 µl sterile PBS to each tube, and incubate the vials at 4°C overnight. Therefore, place them in a slightly tilted horizontal orientation in the fridge with the virus pellet facing down.
  c. The following day, resuspend the virus pellet in the remaining liquid by gentle pipetting and collect all of it in Eppendorf SafeSeal tubes.
    Clean the centrifugation tubes by incubating them for 30 min in Sanosil followed by rinsing with water and preparing them for autoclaving.
  d. Store the concentrated virus at 4°C if used in the next 2 to 3 days or at –80°C for long‐term storage.

#### Raji titration

Determine the amount of virus using Raji titration. All steps should be performed in an appropriate laminar flow hood.

7 Plate 4 × 10^4^ Raji cells in 100 µl R10 in each well of a flat‐bottom 96‐well plate.
8 Inoculate virus on Raji cells. Start with 10 µl concentrate per well, then go to 5, 2.5, 1, 0.5, 0.25, and 0.1 µl.
  Prepare at least duplicates of each condition and include a negative control (Raji cells only, without virus).
9 Fill the wells up to a total of 200 µl with R10 and resuspend the entire well while doing so.
10 Incubate for 48 hr at 37°C in a 5% CO_2_ environment.
11 After incubation, spin down the plate for 5 min at 500 × *g*, 4°C.
12 Remove the supernatant by flipping the plate on a pile of tissues in a BSL‐2 hood.
13 Dilute live/dead Zombi Aqua stain 1:1000 with PBS and add 30 µl of the mixture per well. Incubate in the dark for 10 min at room temperature.
14 Spin down the plate for 5 min at 500 × *g*, 4°C, and flick off the supernatant on a tissue pile in the BSL‐2 hood.
15 Add 80 µl PBS to each well.
16 Resuspend cells, add samples to FACS tubes through cell‐strainer caps and vortex.
17 Acquire samples with a BD FACSCanto flow cytometer.
18 For analysis, use FlowJo and gate on Raji cells, then on single cells, then on the live compartment, and then on GFP^+^ cells.
  Use the negative control to set the gate for GFP^+^ cells.
19 For calculating the viral titer, use those dilutions that give you 1% to 20% GFP^+^ cells. Raji infected units (RIU) per ml concentrate can be calculated as follows:
  a. Calculate the average percentage of GFP^+^ cells for each dilution by calculating the mean of the duplicates.
  b. Multiply by 4 × 10^4^ to determine the total number of eGFP‐expressing Raji cells. These cells are harboring GFP because of viral infection.
  c. To subtract the background, subtract the number of GFP^+^ cells in the uninfected control.
  d. Divide the background‐subtracted total number of GFP^+^ cells by the µl of virus concentrate added and multiply the result by 10^3^. This yields the number of RIU per ml.
  e. For averaging the results, calculate the average of the dilutions that seem alike.

#### Injection of mice

All steps should be performed in an appropriate laminar flow hood. Disinfect the hood and your gloves with Virkon before touching the mice.

20 To perform intraperitoneal (i.p.) injection of mice, calculate the amount of virus concentrate you need to inject the mice with the desired amount of virus (e.g., 10^5^ RIU per mouse).
21 Prepare 0.5‐ml insulin syringes with the planned viral dose, adjusted to a total volume of 100 µl using PBS. Work in a BSL2‐hood and work on ice.
22 For injection in a BLS2 laboratory animal facility, restrain the mice by grabbing them in the neck and pulling back the skin. Disinfect the injection site using 70% ethanol and inject the prepared viral dose into either side of the abdominal cavity.

## Basic Protocol 4 VIRAL LOAD QUANTIFICATION, LYMPHOMA ASSESSMENT, AND IMMUNOHISTOCHEMISTRY AFTER EBV INFECTION OF HUMANIZED MICE

This protocol describes the detection and characterization of EBV infection in humanized mice by viral load quantification based on quantitative PCR (qPCR), macroscopic assessment of lymphoma development, and immunohistochemical analysis of EBV protein expression. Viral loads are quantified from different tissues, e.g., blood or spleen, using TaqMan‐based quantitative PCR targeting the highly repetitive sequence of the BamHI‐W fragment in the EBV genome. Infectious units are determined by comparison to the WHO EBV DNA standard. Lymphoma formation is assessed macroscopically by examination of lymphoid organs, such as spleen or lymph nodes. Lymphoma‐like lesions are typically firm, pale, and nodular, and most frequently found in the spleen of infected humanized mice. Confirmation of EBV‐associated tumorigenesis is achieved by immunohistochemical detection of the latent EBV nuclear antigen 2 (EBNA2). Additionally, immunohistochemistry (IHC) can be used to evaluate the expression of other latent (e.g., LMP1) and lytic (e.g., BZLF1) viral proteins within human CD20^+^ B cells. EBV infection of humanized mice typically results in reproducible detection of EBV DNA in infected tissues, observable lymphoid expansion or lymphoma‐like lesions in a subset of animals, and detectable expression of EBV proteins in human B cells.

### Materials

NSG mice: NOD.Cg‐*Prkdc^scid^ Il2rg^tm1Wjl^*/SzJ (Jackson, strain no. 005557, RRID:IMSR_JAX:005557)
PBS (in house; see Current Protocols, 2006)
NucleoMag Pathogen DNA, RNA, and protein purification (Macherey‐Nagel, 744210.4)
Ficoll‐Paque (GE Healthcare, 17‐5442‐03)
DNeasy Blood & Tissue Kit (Qiagen, 69506)
H_2_O, molecular biology grade (PanReac AppliChem, A7398,0500)
WHO International Standard (NIBSC code: 09/260)
TaqMan Universal PCR Master Mix (Applied Biosystems, 4304437)
Primers (see Table 2)
Formalin solution, 4% (w/v) (Formafix, 20 ml)
Antibodies for IHC:
  Anti‐CD20 antibody (L26; Ventana, 760‐2531)
  Anti‐EBNA2 antibody (PE2; Abcam, ab90543)
OptiView DAB IHC detection kit (Roche, 06396500001)
ultraView Universal AP Red detection kit (Roche, 05269814001)

K2E blood collection tubes (BD Microtainer, 365975)
DxH 500 hematology analyzer (Beckman Coulter)
1.5‐ml SafeSeal tubes (Sarstedt, 7004159)
10‐, 200‐, and 1000‐µl pipettes and tips (Eppendorf)
Tube shaker
NucleoMag SEP Mini magnetic separator (Macherey‐Nagel, 744901)
Analytical balance (Kern‐Sohn, TADJ 200‐4‐A)
Cell strainer, 70‐µm (Falcon, 352350)
Plastic tubes, 50‐ and 15‐ml (Sarstedt, 7510521 and 7510105)
2‐ml syringes, Injekt Luer Solo (B. Braun Medical AG, 4606027V)
Centrifuge, Sorvall ST40R (Thermo Fisher Scientific)
PCR 8‐tube strips (VWR, 732‐1517)
384‐well LightCycler plate (Sarstedt, 72.1985.202)
CFX384 Real‐Time PCR detection system (BioRad, C1000 Touch Thermal Cycler)
TOMO IHC adhesive glass slides (Biosystems, TOM‐11/90)
Software:
  Prism (GraphPad, Version 10.6.0)
  Phenochart (AKOYA Biosciences, Version 1.1.1)
  InForm (AKOYA Biosciences, Version 3.0.0)
  Vectra (AKOYA Biosciences, Version 3.0.7)
Additional basic equipment:
  Herasafe laminar flow hood; class II (Thermo Fisher Scientific)
  Infrared lamp (in house)
  Mouse restrainer (in house)
  Multiwell 24‐well plates (Falcon, 353047)
  Nunc cell culture dish (Thermo Scientific, 153066)
  Omnican Syringe, U‐100 Insulin (Braun, 91511255)
  Pipetgirl (Vitaris)
  5‐ml round‐bottom polystyrene tube with cell strainer cap (Falcon, 352235)
  Scissors (in house)
  Stainless steel surgical blades (Swann‐Morton, 0301)
  3.5‐ml transfer pipette (Sarstedt, 86.1171.001)
  Tweezers (in house)

**Table 2:** Primer and Probes Targeting BamHI W fragment in the EBV Genome

Primer	Sequence
BamHI‐W forward primer	5′‐CTTCTCAGTCCAGCGCGTTT‐3′
BamHI‐W reverse primer	5′‐CAGTGGTCCCCCTCCCTAGA‐3′
BamHI‐W TaqMan probe	5′‐(FAM)‐CGTAAGCCAGACAGCAGCCAATTGTCAG‐(TAMRA)−3′)
GAPDH forward primer	5′‐CAAGGTCATCCATGACAACTTTG‐3′
GAPDH reverse primer	5′‐GGCCATCCACAGTCTTCTGG‐3′
GAPDH TaqMan probe	5′‐(VIC)‐ACCACAGTCCATGCCATCACTGCCA‐(TAMRA)‐3′

#### DNA isolation from the blood

1 Collect 80 µl blood from the tail vein of the mouse or by heart puncture post‐mouse euthanasia. Use a blood collection tube coated with additives to prevent blood clotting.
  Tip: Briefly mix the blood in the tube to completely prevent clotting.
2 Mix 8 µl blood with 8 µl PBS and determine white blood cell count using the DxH 500 hematology analyzer.
3 Take 50 µl of the blood and mix with 150 µl PBS in a 1.5‐ml microcentrifuge tube to isolate DNA.
4 Isolate the DNA using NucleoMag Pathogen viral RNA and DNA purification kit as follows:
  a. Add 20 µl Proteinase K to the sample and mix by pipetting up and down.
  b. Add 180 µl Lysis buffer NPL1 to the mix, mix by pipetting up and down and incubate for 15 min at room temperature with shaking.
  c. Add 20 µl resuspended NucleoMag B‐Beads and 600 µl Binding buffer NPB2 to the lysed sample. Mix by pipetting up and down 6 times and shake for 5 min at room temperature.
  d. Separate the magnetic beads by placing the tubes in the NucleoMag SEP Mini magnetic separator. Wait 2 min until all beads have been attracted to the magnet before discarding the supernatant by pipetting.
  e. Remove the samples from the magnet. Add 600 µl buffer NPW3 and resuspend the beads by shaking until the beads are resuspended completely.
  f. Separate the magnetic beads by placing the tubes in the NucleoMag SEP Mini magnetic separator. Wait 2 min until all beads have been attracted to the magnet before discarding the supernatant by pipetting.
  g. Remove the samples from the magnet. Add 600 µl buffer NPW4 and resuspend the beads by shaking until the beads are resuspended completely.
  h. Separate the magnetic beads by placing the tubes in the NucleoMag SEP Mini magnetic separator. Wait 2 min until all beads have been attracted to the magnet before discarding the supernatant by pipetting.
  i. Remove the samples from the magnet. Add 600 µl of 80% ethanol and resuspend the beads by shaking until the beads are resuspended completely.
  j. Separate the magnetic beads by placing the tubes in the NucleoMag SEP Mini magnetic separator. Wait 2 min until all beads have been attracted to the magnet before discarding the supernatant by pipetting.
  k. Air dry the magnetic bead pellet for 10 min at room temperature.
  l. Remove the samples from the magnet. Add 110 µl of NPE5 to the sample and resuspend beads by shaking 5 min at room temperature.
  m. Separate the magnetic beads by placing the tubes in the NucleoMag SEP Mini magnetic separator. Wait 2 min until all beads have been attracted to the magnet. Transfer the supernatant containing the purified DNA to a new 1.5‐ml microcentrifuge tube.

#### DNA isolation from splenocytes

5 Euthanize EBV‐infected humanized mice in accordance with institutional and ethical guidelines.
6 Perform a midline laparotomy and thoracotomy to expose internal organs under sterile conditions.
7 Harvest the whole spleen of the mouse and put it in PBS on ice.
8 Weigh the spleen using an analytical balance.
9 Mash and filter the spleen through a 70‐µm cell strainer into a 50‐ml tube to obtain a single cell suspension using 10 ml PBS and the pestle of a 2‐ml syringe.
  Tip: Wash the filter in several steps with PBS to obtain a final volume of 10 ml.
10 Isolate mononuclear cells by density gradient centrifugation. Prepare a 15‐ml tube with 4 ml Ficoll‐Paque and carefully layer 10 ml splenocyte suspension on top.
  Tip: Tilt the tube and pipette slowly to not disturb the two forming layers.
11 Centrifuge 25 min at 1000 × *g*, room temperature. Use an acceleration of 4 and deceleration of 1 to preserve the layers during centrifugation.
12 Carefully harvest the cells by inserting a transfer pipette through the upper plasma to the mononuclear cells at the interface and transfer the cells to a new 50‐ml tube.
13 Wash cells by filling the 50‐ml tube with PBS and centrifuge at 5 min at 500 × *g*, 4°C.
14 Resuspend the cell pellet in 1 ml PBS.
15 Add 15 µl of the cell suspension to a PCR tube and determine the white blood cell count using the DxH 500 hematology analyzer.
16 Use 1 × 10^6^ cells to isolate DNA for viral load quantification.
17 Isolate the DNA from splenocytes using the DNeasy Blood & Tissue Kit as follows:
  a. Centrifuge the cells for 5 min at 300 × *g*, 4°C. Resuspend in 200 µl PBS and add 20 µl Proteinase K.
  b. Add 200 µl Buffer AL, mix by vortexing and incubate the samples at 56°C for 10 min.
  c. Add 200 µl of 100% ethanol and mix by vortexing.
  d. Pipet the mix into a DNeasy Mini spin column placed in a 2‐ml collection tube. Centrifuge 1 min at 6000 × *g*, 4°C, and discard the flow‐through.
  e. Add 500 µl Buffer AW1, centrifuge 1 min at 6000 × *g*, 4°C, and discard the flow‐through.
  f. Place the column in a new 2‐ml collection tube and add 500 µl Buffer AW2. Centrifuge 3 min at 20,000 × *g*, 4°C, and discard the flow‐through.
  g. Transfer the spin column to a new 1.5‐ml microcentrifuge tube.
  h. Elute DNA by adding 100 µl nuclease‐free water to the center of the spin column membrane. Incubate for 1 min at room temperature and centrifuge 1 min at 6000 × *g*, 4°C.
  i. Repeat step 18 for increased DNA yield.

#### Viral load quantification by quantitative PCR

18 Prepare the WHO International Standard:
  a. Reconstitute the standard in 1 ml nuclease‐free water to obtain a concentration of 5 × 10^6^ International Units (IU)/ml and leave for a minimum of 20 min with occasional agitation before use.
  b. Mix the standard with blood of a humanized mouse (standard:blood = 1:2).
  c. Prepare a serial dilution to reach a concentration of 5 IU/ml in blood.
    Tip: Prepare a 1:10 dilution series.
  d. Extract DNA using NucleoMag Pathogen viral RNA and DNA purification kit according to the protocol above and elute in 200 µl nuclease‐free NPE5.
    Tip: Prepare your samples and standard on different benches to avoid cross‐contamination. Use filter tips.
19 Prepare the qPCR master mix for samples and standard containing:
  1× TaqMan Universal PCR Master Mix
  10 µM forward primer
  10 µM reverse primer
  10 µM TaqMan probe targeting BamHI‐W repeat region in the EBV genome (Table 2)
  H_2_O, nuclease‐free.

Use primers and probe targeting GAPDH as a positive control for your samples (Table 2).

20 Add 11.25 µl of the master mix to the respective well and mix with 1.25 µl template DNA or standard in a 384‐well plate. Prepare triplicates for each sample or standard.
  Tip: Briefly centrifuge the plate to mix and remove air bubbles.
21 Run the qPCR using the CFX384 Real‐Time detection system and the following protocol as outlined in Table 3.

**Table 3:** Thermal Cycler Program for Viral Load Quantification

Step	Temperature (°C)	Time	No. of cycles
1	50	2 min	1
2	95	10 min	1
3	95	15 s	50
	60	60 s

22 Calculate a standard curve using the serial dilution of the WHO International Standard.
23 Use the standard curve to calculate the equivalent concentration (IU) of your sample.
24 Use white blood cell count of blood samples to calculate IU/ml blood. Use the weight of the spleen as well as the white blood cell count of splenocytes to calculate IU/spleen.

#### Assessment of lymphoma formation

25 Euthanize EBV‐infected humanized mice in accordance with institutional and ethical guidelines.
26 Perform a midline laparotomy and thoracotomy to expose internal organs under sterile conditions.
27 Conduct a macroscopic examination of the spleen for signs of lymphoma formation. Assess enlargement, irregular morphology, and the presence of pale or white nodular masses, which are indicative of lymphoproliferative lesions.
28 Inspect the gastrointestinal tract for visible white or nodular tissue masses. Note that such occurrences are infrequent and less common than splenic abnormalities.
29 Fix the spleen and any other abnormal tissue in 4% formalin and embed in paraffin.
30 Perform immunohistochemistry (IHC) staining for EBNA2 to confirm the presence of EBV‐infected B cells.

#### Immunohistochemical staining of different EBV proteins

31 Cut a section of the spleen, fix in 4% formalin solution, and embed in paraffin.
32 Cut tissue sections of 4 µm from the formalin‐fixed, paraffin‐embedded (FFPE) blocks and mount onto TOMO IHC adhesive glass slides.
33 Perform IHC staining on the BenchMark Ultra automated system using the OptiView DAB IHC and ultraView Universal AP Red detection kit:
  a. Perform CC1 pre‐treatment for 32 min.
  b. Follow the procedure of the instrument User Guide to stain for EBNA2 (1:200 dilution, 60 min incubation) and CD20 (ready‐to‐use, 8 min incubation).
  c. Use diaminobenzidine (DAB) and 3‐amino‐ethylcarbazole (AEC) as chromogens.
  d. Counterstain nuclei with hematoxylin and bluing reagent.

#### Vectra 3 quantitative pathology imaging and analysis

34 Analyze IHC staining at Vectra3 automated quantitative pathology imaging system using the Vectra and InForm software.
35 Scan and acquire whole slide brightfield images of IHC stained splenic tissue using Vectra. Specify in the protocol the brightfield imaging mode and a pixel resolution of 1 µm (10× [10×]).
36 Review the whole slide imagery using Phenochart and annotate regions of interest for multispectral image (MSI) analysis and acquire MSI regions using Vectra. Specify in the protocol a pixel resolution of 0.5 µm (20× [20×]).
37 Use inForm to phenotype cells and measure protein expression in acquired MSI regions: set up an algorithm to recognize and count EBNA2‐ and/or CD20‐positive cells and perform signal evaluation using the algorithm.
38 Check the algorithm's confidence level for a positive signal and count cells with a positive signal with >90% confidence.
39 Determine the number of positive cells per 1 mm^2^.

## Basic Protocol 5 HUMAN T‐CELL RESPONSE ANALYSIS AFTER EBV INFECTION OF HUMANIZED MICE

This protocol is optimized for isolating immune cells obtained from tail vein bleeding or cardiac puncture, as well as from harvested spleens of humanized mice. Following isolation, cells are subjected to surface staining designed to discriminate distinct CD4⁺ T helper subsets, with a particular focus on Th1, Th2, Th9, Th17, and Th22 lineages. Flow cytometric analysis is performed using FlowJo software, which enables precise gating strategies and quantitative assessment of each subset. It is possible to recalculate the total numbers per ml or per total spleen when counting a part of the cell fraction for white blood cells. The staining panel employed in this workflow was designed based on the methodology reported by Sahir et al. (2020), ensuring compatibility with established approaches for dissecting the complexity of human T‐cell differentiation.

### Materials

PBS (in house; see Current Protocols, 2006)
Erythrocyte lysis buffer (see recipe)
Ficoll‐Paque (GE Healthcare, 17‐5442‐03)
Sanosil S010 (Sanosil AG, 12010005CH)
Anti‐human CD45‐BUV395 (BD, 563792)
Anti‐human CD4‐BUV496 (BD, 612936)
Anti‐human CD8‐BUV563 (BD, 612914)
Anti‐human CD14‐BUV661 (BD, 741603)
Anti‐human CD127‐BUV805 (BD, 748486)
Anti‐human CD39‐BV421 (BioLegend, 328214)
Anti‐human CCR6‐SuperBright436 (Thermo Fisher Scientific, 62‐1969‐42)
Anti‐human CD45Ra‐BV510 (BioLegend, 304142)
Anti‐human HLA‐DR‐ BV605 (BD, 562845)
Anti‐human CD137‐BV650 (BioLegend, 309827)
Anti‐human CD25‐BV711 (BioLegend, 356138)
Anti‐human CD3‐BV785 (BioLegend, 317329)
Anti‐human PD1‐FITC (BioLegend, 329904)
Anti‐human CD38‐PerCP (BioLegend, 303520)
Anti‐human CD27‐RB780 (BD, 755842)
Anti‐human CD19‐PE (BioLegend, 302208)
Anti‐human CD197‐PE CF594 (BD, 562381)
Anti‐human CD183‐PE Cy5 (BD, 551128)
Anti‐human CD185‐RY703 (BD, 770737)
Anti‐human CD194‐PE Cy7 (BD, 561034)
Anti‐human CCR10‐APC (BioLegend, 341506)
Anti‐human CD11c‐Alexa 700 (BD, 561352)
Anti‐human CD70‐APC‐Vio 770 (Miltenyi, 130‐104‐360)
Zombie NIR fixable viability kit (BioLegend, 423106)

Paraformaldehyde solution, 4% in PBS (ChemCruz, sc‐281692), optional
Herasafe laminar flow hood; class II (Thermo Fisher Scientific)
K2E Microtainer, blood collection tubes (BD, 365975)
UniCel DxH 520 (Beckman Coulter)
FACS tubes (Sarstedt, 55.1579)
10‐, 20‐, 100‐, 200‐, and 1000‐µl pipettes and tips (Eppendorf)
Vortex mixer
Centrifuge, Sorvall ST40R (Thermo Fisher Scientific)
Corning 96‐well clear polystyrene microplates (Corning, 3894)
Nunc cell culture/Petri dishes (Thermo Fisher Scientific, 153066)
Surgical tweezers (autoclaved)
Cell strainer, 70‐µm (Falcon, 7002108)
2‐ml syringes, Inject Luer Solo (B. Braun Medical AG, 4606027V)
Plastic tubes, 50‐ and 15‐ml (Sarstedt, 7510521 and 7510105)
3.5‐ml transfer pipette (Sarstedt, 86.1171.001)
Cytek Aurora (SpectroFlo v2.2)
FlowJo Software v10.10.0 (BD Life Sciences)
Additional basic equipment:
  Pipetgirl (Vitaris)
  Surgical blades, stainless steel (Swann‐Morton, 0301)
  Filter system, 0.22‐µm for 500 ml (TPP, 7000194)
  Filter system, 0.45‐µm for 500 ml (TPP, 7001474)

#### Blood lysis

All steps should be performed in a BSL‐2 laboratory in an appropriate laminar flow hood.

1 Perform either a tail vein bleeding or a cardiac puncture to obtain blood from the mouse into a blood collection tube (∼100 µl blood should be enough).
2 Use 8 µl of blood diluted with 8 µl PBS to count the white blood cells with the UniCel DxH 520.
3 Label FACS tubes and add 1 ml of 1× erythrocyte lysis buffer per tube.
4 Add 500 µl of 1× erythrocyte lysis buffer directly to the blood collection tube containing the blood.
5 Transfer blood plus erythrocyte lysis buffer from the blood collection tubes into the FACS tubes.
6 Vortex and incubate for exactly 5 min at room temperature.
7 Wash with cold PBS by adding PBS to the top of the tube.
  Washing with cold PBS is vital to stop the lysis. If the lysis is not stopped after 5 min, more leukocytes will die during that process.
8 Centrifuge for 5 min at 500 × g, 4°C.
9 Decant the supernatant and remove last drops by dipping on a paper tissue.
10 Add 500 µl of 1× erythrocyte lysis buffer and resuspend it.
11 Incubate for exactly 5 min at room temperature.
12 Wash with cold PBS by adding PBS to the top of the tube.
13 Centrifuge for 5 min at 500 × g, 4°C.
14 Decant the supernatant and remove last drops by dipping on a paper tissue.
15 Check if most of the red blood cells have been lysed. If not, repeat steps 10 to 14.
16 Resuspend the cells in an appropriate volume; ∼200 µl for staining in a 96‐well plate should be enough.

#### Digesting spleen

17 Weigh spleen in a small Petri dish.
18 Take small piece for histology if needed; weigh the remaining part of the spleen again.
19 Take the rest of the spleen and use surgical tweezers to put it on a 70‐µm cell strainer.
20 Weigh the empty Petri dish to calculate the weight of the total spleen later, without the piece cut away for histology.
21 Mash the spleen through the filter and strain it with the plunger of a 2‐ml syringe and 10 ml PBS into a 50‐ml Falcon tube.
22 Prepare a 15‐ml plastic tube with 4 ml Ficoll‐Paque per spleen. Resuspend the spleen suspension well and layer it very carefully on the 4 ml Ficoll. Tilt the tube and release the suspension very carefully in order not to disturb the Ficoll. Centrifuge for 25 min at 1000 × *g*, room temperature (acceleration 4, deceleration 1).
23 Take the cells layered just above the Ficoll with a transfer pipette without disturbing the Ficoll layer below it. Pipette the cell layer into a new 15‐ml plastic tube and wash with PBS.
  Alternatively, you can also take the whole suspension above the Ficoll and wash it with PBS.
24 After washing, resuspend the pellet in 1 ml PBS and count the white blood cells with the UniCel DxH 520.
25 Dilute cells and distribute cells for FACS staining (0.5 × 10^6^) and viral loads (10^6^ cells) if needed.

#### Surface staining

26 Prepare the staining mix in PBS. Calculate a total volume of 30 ml per sample. The appropriate dilutions are as follows:
  a. CD45‐BUV395, 1:100.
  b. CD4‐BUV496, 1:50.
  c. CD8‐BUV563, 1:100.
  d. CD14‐BUV661, 1:50.
  e. CD127‐BUV805, 1:100.
  f. CD39‐BV421, 1:100.
  g. CCR6‐SuperBright436, 1:50.
  h. CD45Ra‐BV510, 1:100.
  i. HLA‐DR‐ BV605, 1:50.
  j. CD137‐BV650, 1:50.
  k. CD25‐BV711, 1:50.
  l. CD3‐BV785, 1:100.
  m. PD1‐FITC, 1:50.
  n. CD38‐PerCP, 1:50.
  o. CD27‐RB780, 1:200.
  p. CD19‐PE, 1:100.
  q. CD197‐PE CF594, 1:50.
  r. CD183‐PE Cy5, 1:50.
  s. CD185‐RY703, 1:50.
  t. CD194‐PE Cy7, 1:25.
  u. CCR10‐APC, 1:25.
  v. CD11c‐Alexa 700, 1:50.
  w. CD70‐APC‐Vio 770, 1:100.
  x. Zombie NIR, 1:500.
27 Centrifuge the cells 5 min at 500 × *g*, 4°C, and remove the supernatant.
28 Add 30 µl of staining mix per sample. Keep in mind to always have an unstained sample. Incubate for 30 min at 4°C in the dark.
29 Wash the cells with 150 µl PBS and centrifuge for 5 min at 500 × g, 4°C.
30 Re‐suspend cells in 100 µl PBS and proceed to acquire samples on the Aurora.
  If the samples cannot be acquired the same day, the samples can be fixed with 1% PFA for 15 min.

#### FACS analysis in FlowJo

**Figure 4:**
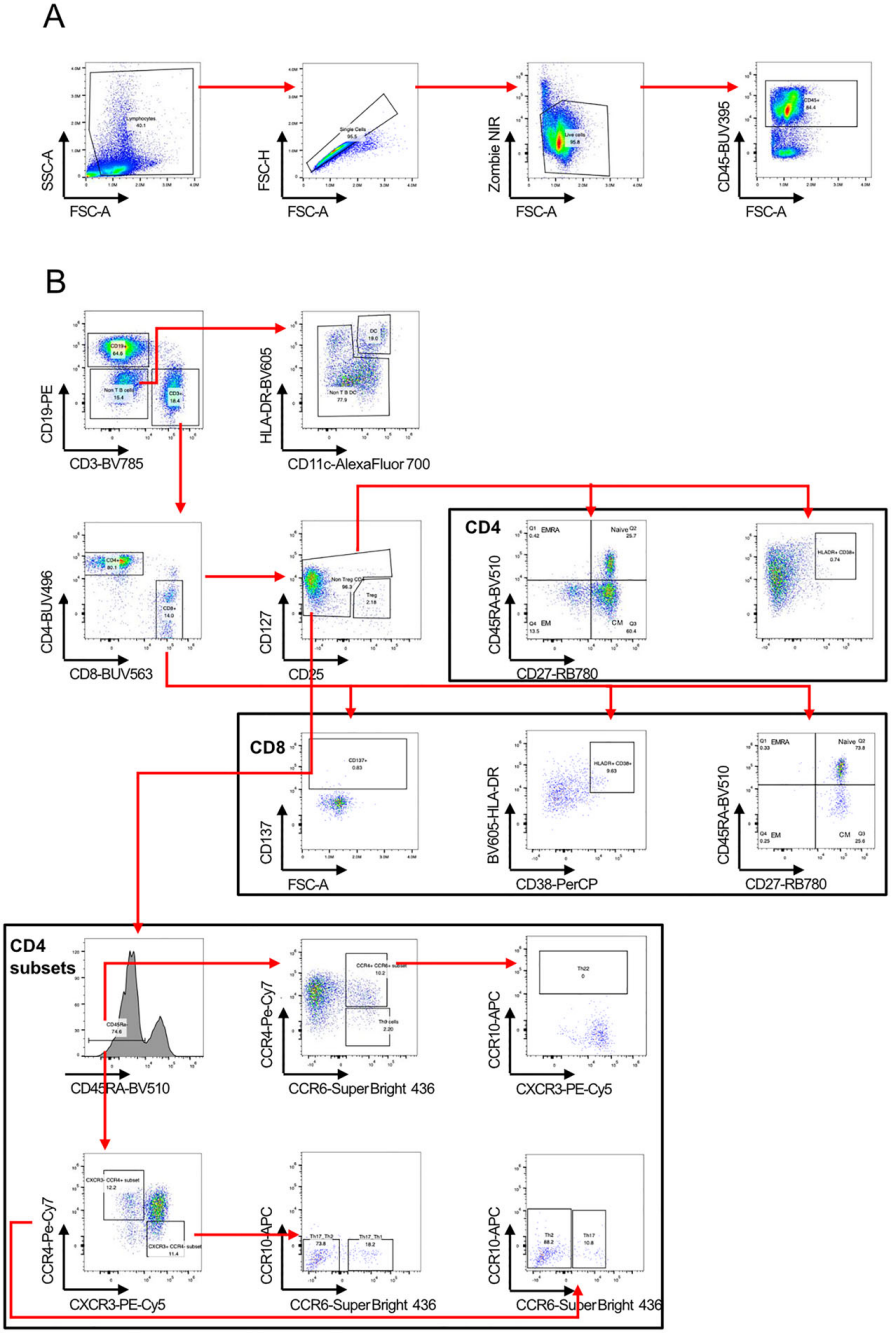
Flow cytometry characterization of spleen cells, isolated from an EBV‐infected humanized mouse. (**A**) Gating strategy for CD45^+^ cells. (**B**) Gating strategy for different CD4^+^ and CD8^+^ subsets as well as B cells and DC cells. EM, effector memory cells; CM, central memory cells; EMRA, effector memory T cells that re‐express CD45RA.

31 Upload all samples to a FlowJo workspace.
32 Follow the gating strategy in Figure 4A. The first gate defines the target cell population (lymphocytes), the second gate excludes duplets, and the third gate excludes dead cells. CD45 is then as a marker to identify leucocytes.

33 For further analysis gating strategy is shown in Figure 4B. CD3 and CD19 are used to identify T cells and B cells, as well as non‐T cells and non‐B cells.
34 The non‐T cells and non‐B cells are then further separated by HLA‐DR and CD11c into non‐T cells, non‐B cells, non‐DC cells, and DC cells.
35 The CD3^+^ cells are split into CD4^+^ and CD8^+^ T cells. Then gate on CD4^+^ T cells to distinguish between CD25^+^CD127^–^ CD4^+^ T‐regulatory cells and the CD25^–^ CD127^±^ CD4^+^ T cells. The second fraction we gate further and use only the CD45RA low cells. These are then separated into two plots:
  a. From the CCR6^+^CCR4^–^ you will get the Th9 subset while from the CCR6^+^ and CCR4^+^ cells you will get the Th22 subset when you use the CCR10^+^ population.
  b. From the CXCR3^–^CCR4^+^ subset you can distinguish two populations the Th2 (CCR6^–^CCR10^–^) and Th17 (CCR6^+^CCR10^–^) subset. From the CXCR3^+^CCR4^–^ subset you can determine Th17_Th1 (CCR6^+^CCR10^–^) subset and the Th17_Th2 (CCR6^–^CCR10^–^) subset.
36 The CD8^+^ as well as the CD25^–^CD127^±^ CD4^+^ T cells can be further separated by two plots. With CD45RA and CD27 we can distinguish naïve (CD27^−^CD45RA^−^), central memory (CM; CD27^+^CD45RA^−^), effector memory (EM; CD27^−^CD45RA^−^), and effector memory T cells that re‐express CD45RA (EMRA; CD27^−^CD45RA^+^). With HLA‐DR, CD38 and CD137 you can check their activation and terminal differentiation status.
37 Gating strategy and antibody panel is based on a published panel by Sahir et al. (2020).

## REAGENTS AND SOLUTIONS

### ACK lysis buffer, 1×

In 0.8 L distilled water, dissolve:
8.023 g ammonium chloride (Sigma‐Aldrich, A9434)
1.001 g potassium bicarbonate (KCl) (Sigma‐Aldrich, 529552)
0.029 g EDTA (ethylenediamine tetraacetic acid) (Sigma‐Aldrich, 03701)
Adjust pH to 7.2 to 7.4 using NaOH or HCl
Add distilled water to bring volume to 1 L
Store up to 6 months at room temperature

### R10 medium

RPMI 1640 (Gibco, 11875093)
10% FCS (Sigma‐Aldrich, S0615‐500ML)
10 U penicillin‐streptomycin (P/S), 10,000 U/ml (Gibco, 15140163)
Store up to 6 months at 4°C

### Digestion mix

8 ml Hanks buffered salt solution (HBSS) +/+, Ca^2+^/Mg^2+^ (Gibco, 7001577)
2 ml of 10 mg/ ml collagenase D (Roche, 7002219) (2 mg/ml final concentration)
10 µl of 20 mg/ml DNase, grade II (Roche, 7002221) (20 µg/ ml final concentration)
Store up to 6 months at 4°C

### Erythrocyte lysis buffer

For a 10× stock solution:
89.9 g NH_4_Cl, ammonium chloride, molecular biology grade (Sigma‐Aldrich, A9434)
10.0 g KHCO_3_, potassium bicarbonate, molecular biology grade (Sigma‐Aldrich, 237205)
370.0 mg EDTA, ethylenediaminetetraacetic acid tetrasodium salt hydrate (Sigma‐Aldrich, 03701)
Dissolve in 1 L ddH_2_O
Adjust pH to 7.3 with NaOH or HCl
Filter through 0.45‐µm filter (TPP, 7001474)
Freeze aliquots and store up to 1 year at –20°C
  Aliquots need to be mixed with ddH_2_O before using, e.g., 5 ml of 10× stock solution with 45 ml ddH_2_O. Always test the stock solution and check for viability of the cells.

### MACS buffer

500 ml PBS (Current Protocols, 2006)
5 ml human serum AB male heat‐inactivated (BioConcept, 2‐13F06‐H)
2 ml of 0.5 M EDTA, pH 8.0 for molecular biology (BioFroxx, 1353ML500)
Filter with a 0.22‐µm filter system (TPP, 7000194)
Aliquot and store up to 2 weeks at 4°C

## COMMENTARY

### Critical Parameters

#### Basic Protocol 1

Minimize travel time and keep human fetal liver tissue properly cooled during transport. Delivery at room temperature significantly reduces cell viability, especially affecting hematopoietic progenitor (CD34⁺) cells.

To ensure the best possible cell viability, adhere to the indicated handing temperatures and incubation times as close as possible. A high cell viability will be crucial for reconstitution success in Basic Protocol 2.

#### Basic Protocol 3

For virus production, a good state of p2089 producer cells is essential. Ensure to split the cells at an adequate frequency so that the cells do not overgrow and that they always have sufficient fresh medium.

Furthermore, the number of seeded Raji cells per well for titration needs to be accurate to make the calculation afterwards as precise as possible.

Only inject mice that show a good reconstitution rate with a human CD45^+^ percentage of ≥10%. Otherwise, you should not use them for a subsequent experiment.

#### Basic Protocol 4

Critical parameters for the experimental procedures include the timing and handling of both blood and spleen samples. Processing of both spleen and blood samples should ideally occur on the same day to ensure optimal cell viability and minimize degradation of labile biomarkers. Cardiac puncture for blood collection must be performed immediately following euthanasia of the mouse to avoid coagulation and hemolysis. The spleen should be placed on ice immediately after dissection to preserve cellular viability.

#### Basic Protocol 5

To ensure the best possible cell viability of the spleen, adhere to the indicated handing temperatures and incubation times as close as possible.

Lysing the blood is important to get rid of the red blood cells. To guarantee that as many lymphocytes as possible survive, complying with the 5‐min lysis time is crucial.

The signal of the individual antibodies can be too high or low, in which case you must titrate the antibodies and use different dilutions.

During the mashing the spleen through the filter, the filter may clog. Make sure to remove all fatty tissue adhering to the spleen before filtering it. In case of a clog, use a new filter.

### Troubleshooting

#### Basic Protocol 1

See Table 4 for a troubleshooting guide for CD34^+^ human hematopoietic progenitor cell isolation and characterization.

**Table 4:** Troubleshooting Guide for CD34^+^ Human Hematopoietic Progenitor Cell Isolation and Characterization

Problem	Possible cause	Solution
MACS column clogged	Too many cells on one column or cell suspension is not homogeneous enough	Use several MACS columns and filter cell suspension again with a 70‐µm cell strainer
Too low or too high antibody signal in flow cytometry	Wrong antibody dilution	Titrate the antibody by using different dilutions; you can use the negative fraction of an old HFL as test samples
Low cell viability	Improper handling temperature or incubation times	Make sure to cool the cells at the relevant steps and adhere to the indicated incubation times as precise as possible

#### Basic Protocol 3

The primary objective of this protocol is to generate a viral yield sufficient for the planned intraperitoneal injection into humanized mice. While the injection procedure is relatively straightforward, challenges may arise during virus production and titration, as outlined in Table 5.

**Table 5:** Troubleshooting Guide for EBV Production and Titration

Problem	Possible cause	Solution
p2089 cells do not attach to the tissue plate after putting them in culture or grow slowly	Medium might not be optimal	Using IMEM medium supplemented with 10% FCS, 1% P/S and 100 µg/ml hygromycin can solve this issue
Low viral yield	Cells were thawed from frozen vials, cultured and transfection was performed shortly afterwards	Passage cells 2‐4 times after putting them in culture before transfecting them
	Cells were in culture for too long	After ∼2 months in culture, cells stop producing high EBV titers, so it is recommended to thaw a new vial
	Cells were too confluent	If the cells are overgrown three days after transfection, seed half of the cells next time (start transfection with a confluency of a bit less than 70%, when in an exponential growth phase)
	p2089 cells do not harbor GFP anymore	Before starting virus production, ensure that your cells still have GFP inside; if not, use a fresh vial of frozen cells
	3 days was not long enough for the cells to produce virus	If the cells do not seem to be destroyed by viral egress 3 days after induction, it might be worth keeping the cells 1‐3 days longer before harvesting the supernatant
	Inefficient switch from latent to lytic cycle	In addition to p509 and p2670, you can add 1.2 µg BRLF1 vector in the transfection step
	Poor transfection efficiency of p2089 cells	Use fresh PEI MAX and vortex vigorously after mixing; verify the quality and concentration of DNA plasmids
No or very low GFP signal in Raji cells	Raji cells were too old or did not grow in optimal conditions	Make sure to split the Raji cells appropriately and do not use ones that were in culture for a long time
	The EBV concentrate was stored too long at 4°C or underwent repeated freeze‐thaw cycles	Use freshly prepared or properly stored virus aliquots at –80°C; avoid repeated freeze‐thaw cycles

### Statistical Analysis

#### Basic Protocol 4

Normality of data distribution is assessed using D'Agostino & Pearson omnibus test. Comparison between two groups of non‐parametric data is performed by unpaired Mann‐Whitney U test. A *p*‐value <.05 is considered statistically significant. All statistical analysis is conducted using GraphPad Prism Software.

### Understanding Results

#### Basic Protocol 1

Figure 2 presents representative flow cytometry plots from the CD34^+^ cell isolation of a human fetal liver. The presented cell frequencies correspond to what can be expected: ≥90% of total cells are alive, and among these, 80% to 90% exhibit the CD34^+^/CD38^–^ phenotype. While the goal of MACS is to exclude CD34^–^, this is rarely achieved in practice as shown in Figure 2B. Nonetheless, successful isolation should demonstrate a clear enrichment of CD34^+^ cells with only minimum accumulation of CD38^+^ cells. If a noticeable enrichment of CD38^+^ cells is detectable, it may indicate compromised tissue quality or suboptimal performance of the CD34 isolation kit.

#### Basic Protocol 2

**Figure 5:**
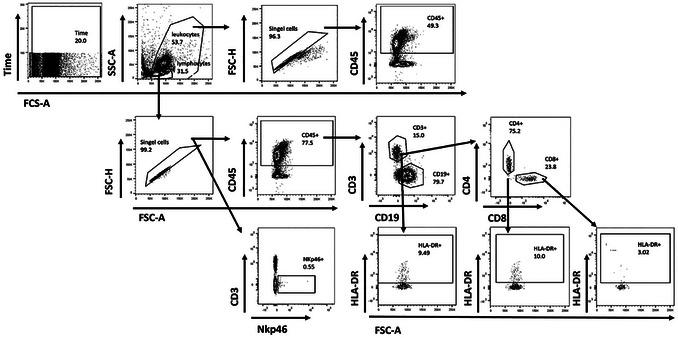
Gating strategy for human immune cell reconstitution in the peripheral blood of humanized NSG mice. Flow cytometry gating strategy of one exemplary blood sample from a reconstituted mouse (FlowJo). Percent of parent was used for calculations of mean and standard deviation.

**Figure 6:**
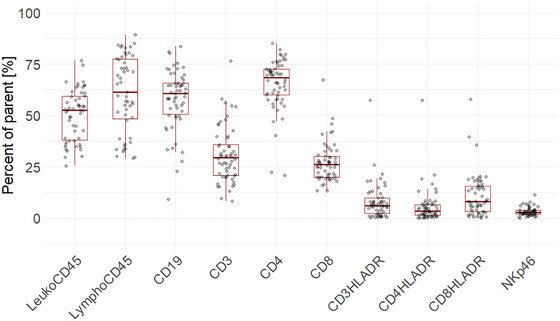
Phenotypic characterization of reconstituted NSG mice with HFL‐derived CD34^+^ cells. Flow cytometry analysis of reconstituted NSG mice after 3 months. Cells were stained for CD45, CD19, CD3, CD4, CD8, Nkp46, and HLA‐DR. The respective gating strategy is shown in Figure 2. Cell debris and doublets were excluded from the analysis based on scatter signals. Mean values of 58 reconstituted NSG cohorts are shown as dots. Visualization of IQR with boxplots. Outliers are included.

Figure 5 presents the gating strategy for flow cytometry plots for the characterization of humanized mice 12 weeks after reconstitution. Reconstitution is considered successful when there are >10% human CD45^+^ cells in the blood. Presented cell frequencies in Figure 6 correspond to what can be expected: at least 40% to 60% of total cells are CD45^+^. B cells (CD19^+^) show a higher prevalence, ranging from 50% to 65% of CD45^+^ cells, compared to T cells (CD3^+^), which ranged from 25% to 35% of CD45^+^ cells. CD4^+^ T cells constitute most T cells in the blood, comprising an average of ∼70% of the total T‐cell population, while CD8^+^ T cells only constitute ∼25%. Additionally, HLA‐DR expression, a marker indicative of T‐cell activation, should be <10%.

#### Basic Protocol 3

**Figure 7:**
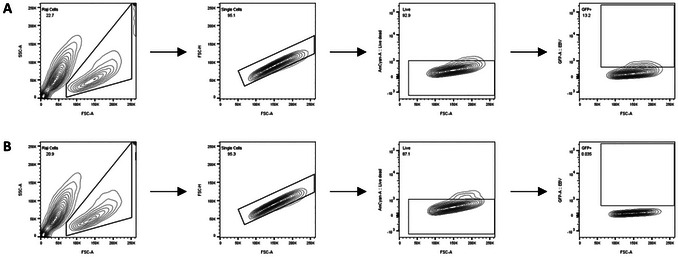
Flow cytometry gating to detect GFP^+^ Raji cells. Sequential gating for GFP^+^ Raji cells incubated for 48 hr with 10 µl EBV (**A**) or without the addition of EBV (**B**) as described above. After gating for lymphocytes, we gated for single cells, live cells, and GFP^+^ cells. The numbers shown within the plots represent the percentage of the frequency of parents of the plotted subpopulations.

In the exemplary data depicted in Figure 7, the frequency of grandparents of GFP^+^ cells was 12.3% for Raji cells incubated with 10 µl EBV concentrate (Fig. 7A). The 0.3% of GFP^+^ cells in the uninfected control are considered background signal (Fig. 7B). After subtracting this background, this resembles a viral titer of ∼4.8 × 10^6^ RIU/ml for the concentrate. The viral yield to expect from EBV virus production, as explained above, highly depends on the amount of transfected producer cells. This viral titer of ∼5 × 10^6^ RIU/ml was obtained by transfecting five 15‐cm dishes, which can be considered a successful virus production.

Successful infection of Raji cells with EBV is indicated by a distinct population of GFP^+^ Raji cells, representing 1% to 20% of the total live gate, with percentages increasing proportionally to the viral concentration. Low infection efficiency may result in a weak or diffuse GFP signal, which may require repeating the titration using freshly prepared virus.

In vivo, a dose of 10⁵ RIU per mouse typically results in detectable EBV DNA in peripheral blood (see Basic Protocol 4) within ∼3 weeks post‐injection, depending on their reconstitution level. Lower infection rates may reflect insufficient dose or poor immune reconstitution.

#### Basic Protocol 4

**Figure 8:**
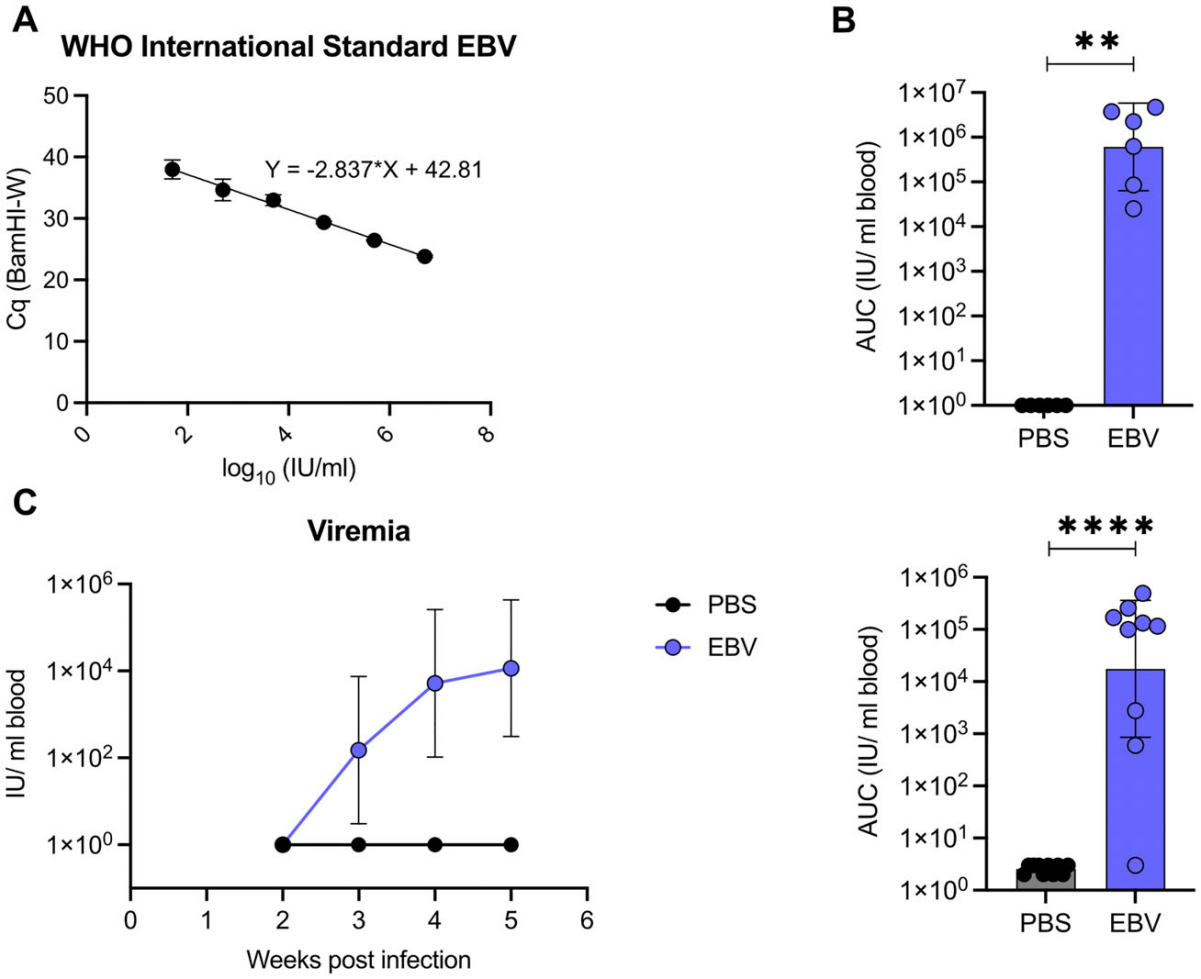
Quantification of viral loads in blood and spleen from EBV‐infected humanized mice. (**A**) Standard curve generated using different dilutions of the WHO International Standard in TaqMan qPCR targeting. Linear regression analysis was performed on C_q_ (mean ± *SD*) values against the log_10_‐transformed input IU of the standard. IU of EBV viral loads per spleen [geometric mean ± 95% confidence interval (CI)] (**B**) and weekly blood viral loads (IU/ml blood; geometric mean ± 95% CI) (**C**) of EBV‐infected and ‐uninfected (PBS) mice. Area under the curve (AUC) was calculated for each individual mouse. Comparison between groups was performed by unpaired Mann‐Whitney U test. **p < .01, ****p < .0001.

Figure 8 presents the quantification of EBV viral loads from blood and spleen from infected humanized mice by TaqMan qPCR targeting the BamHI‐W repeat region. The standard curve was generated using the WHO International Standard and linear regression of Cq values against the log_10_‐transformed input IU of the standard. This standard curve was used to calculate IU per ml blood or spleen from infected humanized mice.

**Figure 9:**
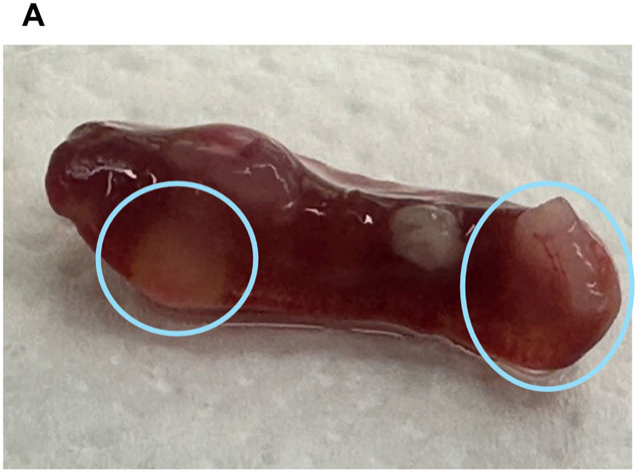
Spleen isolated from an EBV‐infected humanized mouse. Enlarged, white nodular masses are highlighted by blue circles.

Figure 9 displays a spleen isolated from an EBV‐infected humanized mouse. Enlarged, white nodular masses (encircled regions) indicate EBV‐driven lymphoid expansions. The presence of EBV requires further confirmation by immunohistochemical analysis.

**Figure 10:**
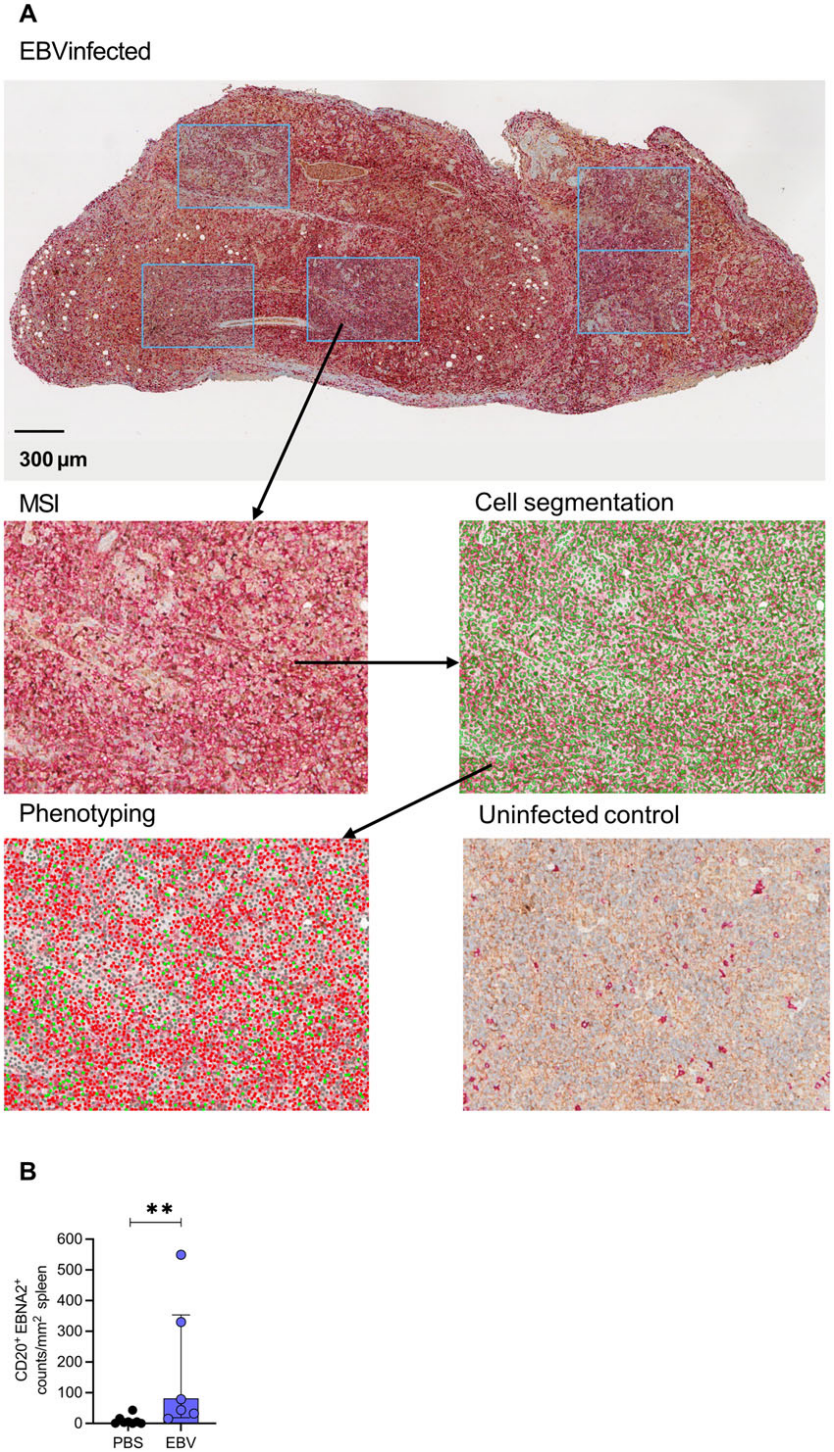
(**A**) Immunohistochemical staining of CD20 and EBNA2 in a spleen isolated from an EBV‐infected humanized mouse. Annotations of multispectral image (MSI) are indicated in blue quadrants. Using the MSI, the algorithm was trained to recognize individual cells, shown in the single‐cell segmentation image. Single cells are highlighted in green. Subsequent phenotyping classified cells as CD20⁺EBNA2⁺ (green), CD20⁺ (red), or CD20^–^EBNA2^–^ (grey). (**B**) Quantification of CD20^+^EBNA2^+^ splenocytes in EBV‐infected (blue) and uninfected (PBS; black) humanized mice. Comparison between the groups was performed by unpaired Mann‐Whitney U test. **p < .01.

Figure 10 illustrates the analysis of brightfield images of a spleen from an EBV‐infected humanized mouse using Vectra and InForm software. The image analysis was initiated by training the algorithm to identify individual cells (cell segmentation). In the subsequent phenotyping step, the algorithm was trained to distinguish between positive (CD20^+^EBNA2^+^ in green, CD20^+^ in red) and negative cells (in gray). Once trained, the algorithm was applied to analyze spleen sections from multiple animals in a standardized and automated manner.

#### Basic Protocol 5

Figure 4 presents representative flow cytometry plots from the CD45^+^ cells isolated of a spleen or of blood from a humanized mouse. The presented frequencies for CD19 or CD3 cells can vary strongly between the individual mice even when injected with the same stem cells. The individual amount of the different T‐cell subsets depends on activation and infection, and which organ is used to stain the cells. In the spleen, frequencies are generally higher, and more cells can be recovered compared to the blood. In the example shown, the largest part are CD19^+^ cells while the CD3^+^ cells are mainly CD4^+^ T cells. Within the CD4^+^ T‐cell subsets mainly Th2 and Th17 cells are abundant, while there are almost no Th9 and Th22 cells.

### Time Considerations

#### Basic Protocol 1

Basic Protocol 1 requires 1 full working day (8 to 9 hr). Ideally, schedule tissue delivery for the morning. Prepare the laminar flow hood and all reagents for the digestion mix in advance to save time. Below is a rough time estimation for each substep.

Digestion and PBMC isolation: ∼2.5 to 3 hr
MACS: 1.5 to 2 hr
Cell freezing: 30 min
Flow cytometry staining: 1 hr
Flow cytometry acquisition and analysis: 2 hr

To save time, prepare reagents for upcoming steps during incubations and freeze cells while performing flow cytometry staining. Only perform steps in parallel if you are familiar with the techniques in the protocol.

#### Basic Protocol 3

The expansion of 2089 producer cells to a scale sufficient for the intended virus yield typically requires 2 to 3 weeks. Once the culture reaches the required scale, virus production takes 6 days, including transfection, harvest, and concentration. Raji titration and flow cytometry acquisition typically require up to 3 days, while data analysis can be completed in <1 hr. Intraperitoneal injection of mice is rapid, taking <1 min per animal.

### Author Contributions

**Saskia Gertrud von Boxberg**: Data curation; formal analysis; investigation; methodology; validation; visualization; writing—review and editing. **Kristin Gehrmann**: Data curation; formal analysis; investigation; methodology; validation; visualization; writing—original draft. **Svenja Luisa Nopper**: Data curation; formal analysis; investigation; methodology; validation; visualization; writing—original draft. **Lucas Romann**: Data curation; formal analysis; investigation; methodology; validation; visualization; writing—original draft. **Svenja Kösegi**: Data curation; formal analysis; investigation; methodology; validation; visualization; writing—original draft. **Christian Münz**: Conceptualization; funding acquisition; project administration; supervision; writing—original draft; writing—review and editing.

### Conflict of Interest

The authors declare no conflict of interest.

## Data Availability

Data available on request from the authors.
